# Clinical consequences of road traffic injuries among the elderly in Japan

**DOI:** 10.1186/1471-2458-10-375

**Published:** 2010-06-28

**Authors:** Takashi Nagata, Hajime Uno, Melissa J Perry

**Affiliations:** 1 Department of Emergency Medicine, Himeno Hospital, Fukuoka, Japan; 2 Takemi Program, Department of International Health, Harvard School of Public Health, Boston, MA, USA; 3 Department of Biostatistics and Computational Biology, Dana-Farber Cancer Institute, Boston, MA, USA; 4 Department of Environmental Health, Harvard School of Public Health, Boston, MA, USA

## Abstract

**Background:**

Road traffic injuries among the elderly have recently become a public health issue; therefore, we investigated the clinical characteristics of such injuries among the elderly in Japan.

**Methods:**

A retrospective study was performed using data from a medium-sized hospital emergency department. Data were extracted from medical records for one year, and patients were categorized into groups ages 18-64, 65-74 and 75+. Variables included demographic characteristics, injury circumstances, and nature of injury. Univariate and bivariate descriptive statistical analyses were performed, and multivariate logistic regression was used to evaluate injury severity and hospital admission by age groups.

**Results:**

A total of 1,656 patients were studied. Patients aged 65+ had more chest wall injury, intracranial injury, lower extremity fracture, and intrathoracic injury than patients aged 18-64.

**Conclusions:**

Injury circumstances and nature of injuries associated with traffic incidents showed different patterns by age groups, particularly among the elderly.

## Background

Most industrialized countries are rapidly becoming aging societies, and road traffic injuries among the elderly are therefore becoming a major public health issue [1,2]. In the United States, the Centers for Disease Control and Prevention (CDC) has estimated that about 26 million licensed drivers are aged 65 and over (75% of people aged 65 and over are licensed), and 40 million people in this age group will be licensed by 2020. In 1999 alone, more than 7,000 Americans 65 and older died, and another 246,000 suffered non-fatal injuries, resulting from motor vehicle crashes. Thus, traffic injuries among the elderly are becoming a serious social problem in the United States [3]. Elderly people are involved in 40% of fatal traffic injuries in the European Union [4]. Further, pedestrian injuries among the elderly exacerbate the issue. A report from the Organization for Economic Co-operations and Development (OECD) showed that in several European countries, pedestrians aged 65 years and older, though representing only 15% of the total population, accounted for 45% of all pedestrian fatalities [5]. These data indicate that traffic injuries among the elderly are a serious global problem that warrants further investigation.

Japan has a large elderly population, and life expectancy for women is the longest in the world (Female: 86 years, Male: 79.2 years in 2007) [6]. The percent of the population aged 65 and older in Japan was 22.1% in 2008. Importantly, road traffic safety risks to the older population in Japan appear to be considerable. The most recent National Police Agency (NPA) report in Japan showed that about 48 of total traffic fatalities and about 69% of total pedestrian fatalities occurred in people aged 65 and older. The NPA, Japan, has attempted traffic safety promotion programs targeting people 65 and over; however, they have yet to achieve major reductions in traffic incidents [7,8].

Hospital emergency department-based injury registries are an important front line for conducting injury epidemiology studies. In addition to traffic injury research, these registries are a source of data for numerous other types of injuries systematically tracked in emergency departments, including occupational injuries, intentional injuries such as suicide, and pediatric injuries [9-13]. In Japan, agencies such as local police units, hospitals, health insurance companies, local government offices, and academic associations have established injury databases, and the national police agency manages a centralized injury database. However, these databases are not well linked to each other [7,8]. To date, few of the published injury registry studies have been based in a hospital emergency department setting in Japan [14].

The primary objective of this study was to describe the clinical consequences, i.e., injury severity, hospital admission, and length of stay, of traffic injuries among the elderly at one hospital in Japan. The study was based in a medium-sized emergency department in Japan, and injury visits were tracked and analyzed by comparing occurrence injury patterns of the elderly (65 years and older) and non-elderly with patient demographics, injury circumstances, and nature of injury. We hypothesized that differences in the clinical consequences of road traffic injuries would exist between the elderly and younger people who visited the emergency department. Of particular interest were the differences between those 65-74 years old and those in the 75+ age group.

## Methods

### Sample, Setting, and Data Collection

Two data sources were used. First, basic characteristics of road traffic incidents in Japan were taken from the data of the NPA, Japan, as a public domain. Number of road traffic incidents, percentage of males, mechanism of injuries, and injury severity (fatalities, severe injuries, and injuries) were available. The NPA, Japan, defines severe injury as those needing in-hospital and/or out-patient care for more than 30 days after injury.

Second, data were collected retrospectively from April 1, 2003 through March 31, 2004 at Saint Mary's Hospital, Kurume, Japan. Kurume City (population of approximately 300,000) is located in Kyushu in southwestern Japan. The main industry of Kurume is agriculture, and the economic status of the population in this area is within the average range in Japan. Saint Mary's Hospital has 1,400 beds. The hospital works as a referral hospital; moderate to severely injured patients are often transferred there from local community hospitals. The population in the catchment area of this hospital was estimated to be about 860,000. Other hospitals in the area also receive injured patients. Saint Mary's Hospital is a Level 2 facility handling injuries of minor to moderate severity; life-threatening injuries are usually transferred to a Level 1 hospital. Annual patient volume in the emergency department (ED) was about 60,000 during the study period, and about 50% of the total emergency cases were transferred in from outside Kurume City. Injury-related ED visits comprised about 1/6 of the total annual patient load, and traffic-related visits were about 1/5 of all injury visits.

Study personnel were trained to extract injury data from hospital records, including admission and billing records; to assign International Classification of Diseases, Tenth Revision (ICD-10) diagnosis and injury cause codes; and to transfer this information onto study data forms.

The total number of injury-related ED visits was 10,621 in 2003. Of those, 2,122 were traffic-related injury visits among ages 0 to 94 years. The inclusion criteria for a traffic-related injury visit was defined as any patient who was injured in a traffic-related incident as indicated by an ICD-10 code (V01-89) and came to the hospital as a walk-in or by ambulance. Of the 2,122 traffic-related injury patients, 78 were removed as misclassification (33), data missing (20), and emergency department repeat visits (25); 388 cases who were 17 years or younger were also removed. This study included only adult traffic injuries among the 1,656 individuals who were 18 years and older.

### Measures

Data collected from each injury case included: demographic, injury circumstance, and nature of injury; personal identifiers such as name and patient ID were removed. Demographic data included age, gender, and current address. Injury circumstance data included date of injury, description of traffic injury, hospital transfer circumstance (ambulance or walk-in, with walk-in indicating transport other than by ambulance), time of hospital visit, and type of health insurance. Time of ED visit was categorized as: daytime (08:30 to 16:59), evening (17:00 to 23:59), or night (0:00 to 8:29); and weekday (Monday to Friday) or weekend (Saturday and Sunday). Health insurance was categorized according to the five types of payment in Japan: national mandatory car insurance, social health insurance, national health insurance, occupational health insurance, and self pay.

Mechanism of injury at the time of crash was classified into five categories: automobile, bicycle, motorcycle, pedestrian, and other. People injured in automobile crashes include drivers and occupants, but not pedestrians hit by an automobile. Pedestrian injuries were coded when they were caused by a collision with a motor vehicle. "Other" referred to traffic injuries that could not be classified (e.g., falling horse).

Nature of injury described location on the body. ICD-10 diagnosis codes for anatomic injury were assigned to each injury. One injury circumstance and one injury diagnosis were coded for all visits. Secondary and tertiary diagnoses were also coded according to the patients' clinical diagnosis. A total of 999 visits included a secondary diagnosis code and 235 included a third diagnosis code. Sixty-eight visits had missing injury circumstance or injury diagnosis codes.

The Abbreviated Injury Scale (AIS) was also assigned to each ICD-10 diagnosis, and an Injury Severity Score (ISS) was calculated [15]. Based on prior studies, two categories were created to investigate the associations with diagnosis group, injury severity and hospital admission: slight and moderate (ISS ≤ 8) and severe (ISS ≥ 9) [16,17]. Three categories were used (slight ISS ≤ 3, moderate ISS 4 - 8, and severe ISS ≥ 9) to compare demographic characteristics, injury circumstances, and nature of injury.

Nature of injury data also included fatalities, admission (yes/no), intensive care unit (ICU) admission (yes/no), treatment (type of surgery), and length of hospital stay (days). For type of operation/surgical procedure, minor surgery was defined as that performed in the ED (e.g., wound suture, splinting). Elective surgery was defined as a surgical procedure done electively after admission. Emergency surgery was defined as an urgent surgical intervention after being transferred directly from the ED to the operating room in the case of life-threatening situations. Clinical information regarding pre-existing diseases and ICU stay was not included in the dataset.

### Statistical Analysis

Injuries among patients aged 65 and older were classified as elderly-related injuries. Three age groups, aged 18-64 (defined as Group 1), 65-74 (defined as Group 2), and 75 and older (defined as Group 3), were compared in the analysis.

Differences in demographic characteristics, injury circumstance, and nature of traffic injury cases by age group were compared by chi-squared tests. This method was applied to the comparison between the three age groups: Group 1 vs. Group 2, Group 1 vs. Group 3, and Group 2 vs. Group 3. Because of multiple comparisons, a Bonferroni correction was applied, such that the typical significance level of p-value ≤ 0.05 was adjusted to p-value ≤ 0.01 (0.05/3 = 0.016).

The prevalence ratio (PR) was used to evaluate the association of age with injury circumstances and nature. Reports in the epidemiology literature have indicated that the PR is preferable to the odds ratio for the analysis of cross-sectional data, because the odds ratio can overestimate an association, especially if the condition of interest is common [18,19]. The prevalence ratio of elderly traffic injuries associated with different patient demographic characteristics was calculated as the ratio of the prevalence in the age group of interest, compared with the corresponding prevalence in the age reference category. PRs together with 95% confidence intervals (CI) were calculated using logistic regression analysis. Crude and adjusted estimates were calculated for each separate factor [18,19].

The prevalence ratio of clinical injury diagnosis was calculated in the elderly (aged 65+), compared with the corresponding prevalence in the reference category (aged 18-64).

Multivariate logistic regression methods were performed to construct models involving factors associated with injury severity (ISS ≥ 9) and hospital admission. Covariates for the final multivariate logistic regression models were selected using a backward method. Evaluation of the goodness-of-fit of the final models was done using the Hosmer-Lemeshow test [20]. For the final models, the area under the receiver operating characteristic (ROC) curve was calculated.

All statistical analysis was performed using SAS software, version 9.1. Statistical significance was accepted at a p-value less than 0.05, except where the Bonferroni correction was used.

### Ethical considerations

Ethical approval was obtained from the Institutional Review Board at Harvard School of Public Health, US, and Saint Mary's Hospital, Japan.

## Results

Table 1 shows the demographic characteristics and injury circumstance of road traffic injuries based on the data of National Police Agency, Japan, according to three age categories and severities. The percentages of severe injuries and total injuries among patients aged 65-74 and 75+ were higher than those of patients aged 18-64.

**Table 1:** Basic characteristics of national level road traffic injuries in 2005, Japan

Age (years)	18 - 64	65 - 74	75+
Group	1	2	3
Population	80,271,702	14,070,107	11,576,545

Cases (percentage of the population)			
Fatality	3668 (0.005%)	1240 (0.009%)	1572 (0.014%)
Severe injuries	43336 (0.054%)	10372 (0.074%)	7887 (0.068%)
All injuries	904086 (1.13%)	86968 (0.618%)	42298 (0.365%)

Gender (% of male)			
Fatality	80%	58%	51%
Severe injuries	63%	44%	47%
All injuries	55%	50%	50%

Mechanism of injuries (%)			
Fatality			
Automobile	53%	29%	20%
Pedestrian	18%	40%	52%
Bicycle	8%	18%	17%
Motorcycle	21%	13%	10%
Others	0%	0%	0%

Severe injuries			
Automobile	38%	27%	22%
Pedestrian	10%	23%	34%
Bicycle	14%	26%	26%
Motorcycle	37%	24%	18%
Others	0%	0%	0%

All injuries			
Automobile	69%	52%	39%
Pedestrian	4%	13%	24%
Bicycle	11%	23%	25%
Motorcycle	15%	13%	12%
Others	0%	0%	0%

Of the 1,656 traffic injuries, 88% involved people aged 18-64 (Group 1), 7% involved those aged 65-74 (Group 2), and 5% involved those 75 and older (Group 3; see Table 2). Fewer than 2% of all patients were covered by occupational health insurance. Males were more prevalent in Group 1 compared to Group 2. Orthopedic surgery was less frequent in Group 3 than Group 1. Emergency department visits in the morning were more frequent in Groups 2&3 than in Group 1.

**Table 2:** Demographic Characteristics and Injury Circumstance among Traffic Injury Cases (N = 1,656) by age groups, Japan, 2003

Group	1	2	3
Age (years)	18 - 64	65 - 74	75 +
Cases	1459	119	78

Gender (% males)	56.8 *	38.7	48.7
Address (% outside Kurume)	41.9	34.5	35.9

Transfer Circumstance (%)			
Ambulance	47.4 *	73.1	82.1 ***

Payment Type (%)			
Mandatory car insurance	69.5 *	52.1	57.7
Social insurance	18.5	16.8	10.3
National insurance	5.7 *	26.9	29.5 ***
Occupational insurance	1.9	0.0	0.0
Self pay	4.4	4.2	2.6

Medical Service (%)			
Orthopedic	80.3	72.3	57.7 ***
Neurosurgery	8.7	14.3	19.2 ***
Plastic surgery	5.6	2.5	9.0
General surgery	4.7	9.2	11.5 ***
Others	0.6	1.7	2.6

Time of ED visit (%)			
08:30 - 16:59	32.9 *	53.8	57.7 ***
17:00 - 23:59	43.7	37.0	30.8
00:00 - 08:29	23.4 *	9.2	11.5

Weekend (%)	35.6	37.0	26.9

Mechanism of Injury (%)			
Automobile	68.0 *	38.7	37.2 ***
Pedestrian	4.7 *	21.9	20.5 ***
Bicycle	8.8 *	25.2	29.5 ***
Motorcycle	16.5	11.8	11.5
Others	2.1	2.5	1.3

* group 1 is significantly different (P ≤ 0.01) from group 2

** group 2 is significantly different (P ≤ 0.01) from group 3

*** group 3 is significantly different (P ≤ 0.01) from group 1

There was no significant difference between Groups 2&3 in any category. Groups 2&3 were significantly more frequently transferred by ambulance than Group 1. Regarding mechanism of injury, about 70% of Group 1 was injured in automobile incidents (drivers or occupants), significantly more than in Groups 2&3. Pedestrian and bicycle injuries were significantly more common in Groups 2&3 compared to Group 1.

We further analyzed the nature of injuries related to traffic incidents (Table 3). Among the three age groups, neck injuries were significantly less common in Groups 2&3 compared to Group 1. Chest injuries were more prevalent in Group 2. Prevalence of hospital admission and ICU admission were higher in Groups 2&3 compared to Group 1. Injury severity, elective surgery, and hospital admissions greater than 28 days were also significantly higher in Groups 2&3 compared to Group 1. The mean ISS in this study population was 3.2 and the range was 1-50. The mean ISS for the 18-64 was 2.8 (range: 1-50); 65-74 was 5.9 (range: 1-34); and 75+ was 6.1 (range: 1-34).

**Table 3:** Nature of Road Traffic Injury Cases (N = 1,656) by age groups, Japan, 2003

Group	1	2	3
Age (years)	18 - 64	65 - 74	75 +
Cases	1459	119	78

Anatomic region (%)			
Neck	45.4 *	18.5	18.9 ***
Head	16.6	21.0	30.8 ***
Chest	9.9 *	28.6 **	10.3
Abdominal	6.1	8.4	9.0
Upper limb	10.6	5.9	11.5
Lower limb	11.5	17.7	20.5

Outcome (% mortality)	0.9	2.5	6.4***

Hospital admission (%)	16.5 *	47.1	48.7 ***

ICU admission (%)	4.7 *	16.8	17.9 ***

Treatment (%)			
None	86.4 *	68.9	70.5 ***
Minor surgery	7.3	12.6	16.7 ***
Elective surgery	5 *	14.3	7.7
Emergency surgery	1.4	4.2	5.1

Injury Severity Score (ISS) (%)			
Slight(ISS ≤ 3)	82.3 *	52.9	52.6 ***
Moderate(ISS 4 - 8)	10.4	14.3	19.2
Severe(≥ 9)	7.3 *	32.8	19.2 ***

Admission days (%)			
Return home	83.5 *	52.9	51.3 ***
Less than 7 days	4.0	6.7	10.3
for 7 - 13 days	3.1	6.7	9.0
for 14 - 27 days	3.9	12.5	6.5
more than 28 days	5.6 *	21.0	23.1***

* group 1 is significantly different (P ≤ 0.01) from group 2

** group 2 is significantly different (P ≤ 0.01) from group 3

*** group 3 is significantly different (P ≤ 0.01) from group 1

When the injury diagnosis was analyzed by age group, the leading four traffic injuries in patients aged 65 years and older were chest wall injury, neck sprain and strain, intracranial injury, and lower extremity fracture (Table 4). The leading traffic injuries with high PRs were chest wall injury, intracranial injury, lower extremity fracture, and intrathoracic injury. Excluding neck sprain and strain (S134), intracranial injury, lower extremity fracture, and intrathoracic organ injury were the leading traffic injuries with high PRs.

**Table 4:** Diagnosis group of ≥ 65 years vs. 18 - 64 years old (N = 1,656) in Japan, 2003

	Age					
	≥ 65		18 - 64			
				
	n	**(%)**^**a**^	n	**(%)**^**a**^		
	197	11.9%	1459	88.1%		
Location of injury (ICD-10)	n	**(%)**^**b**^	n	**(%)**^**b**^	PR (95%CI)	PR (95%CI) S134 removal analysis
Chest wall injury (S20-25, S28-29)	32	16.2%	128	8.8%	1.85 (1.30 - 2.65)	1.23 (0.87 - 1.74)
Neck sprain and strain (S134)	31	15.7%	643	44.1%	0.36 (0.26 - 0.50)	-
Intracranial injury (S06)	26	13.2%	70	4.8%	2.75 (1.80 - 4.20)	1.82 (1.20 - 2.77)
Lower extremity fracture (S72, S82, S92)	24	12.2%	57	3.9%	3.12 (1.98 - 4.90)	2.07 (1.32 - 3.23)
Mild head injury, face injury (S00-S02)	17	8.6%	128	8.8%	0.98 (0.61 - 1.59)	0.65 (0.40 - 1.05)
Mild abdominal and pelvic injury (S30-S35, S38-39)	15	7.6%	76	5.2%	1.46 (0.86 - 2.49)	0.97 (0.57 - 1.65)
Lower extremity injury (S70-99)	13	6.6%	110	7.5%	0.88 (0.50 - 1.53)	0.58 (0.34 - 1.01)
Other neck injury (S01-S19)	11	5.6%	63	4.3%	1.29 (0.70 - 2.41)	0.86 (0.46 - 1.59)
Intrathoracic organ injury (S26,27)	10	5.1%	17	1.2%	4.36 (2.02 - 9.38)	2.89 (1.35 - 6.20)
Upper extremity fracture (S42, S52, S62)	7	3.6%	35	2.4%	1.48 (0.67 - 3.29)	0.98 (0.44 - 2.17)
Upper extremity injury (S40-S69)	9	4.6%	119	8.2%	0.56 (0.29 - 1.09)	0.37 (0.19 - 0.72)
Abdominal organ injury (S36,37)	2	1.0%	13	0.9%	1.14 (0.260 - 5.01)	0.76 (0.17 - 3.32)

PR = Prevalence Ratio

^a^Percent of total, ^b^Column percent

For hospital admission (Table 5), the PR in ages 65-74 (PR = 2.90, CI = 1.77-4.74) was higher than the PR in age 75+ (PR = 2.57, CI = 1.46-4.55). For injury severity (Table 6), the PR in ages 65-74 (PR = 3.96, CI = 2.35-6.65) was higher than the PR in age 75+ (PR = 2.44, CI = 1.32-4.51).

**Table 5:** Prevalence Ratios estimating factors associated with hospital admission in a multivariate logistic regression model (N = 1,656), Japan, 2003

Factors	N	PR adj.	95% CI	
Gender				
Females	744	1.00		
Males	912	1.23	0.87	1.53

Age				
18 - 64	1459	1.00		
65 - 74	119	2.90	1.77	4.74
75 -	78	2.57	1.46	4.55

Mechanism of Injury				
Automobile	1067	1.00		
Bicycle	182	5.13	3.22	8.17
Motorcycle	263	5.15	3.47	7.64
Pedestrian	111	5.07	3.00	8.57
Others	33	3.12	1.25	7.79

Address				
in Kurume	976	1.00		
outside Kurume	680	2.78	2.02	3.83

Transfer				
Walk-in	813	1.00		
Ambulance	843	20.40	7.79	12.49

Weekend				
Yes	585	1.00		
No	1071	1.73	1.25	2.40

PR adj: Adjusted Prevalence Ratios

The area under ROC curve = 0.875

P-value of the goodness of the fit = 0.389

CI: Confidence Intervals

**Table 6:** Prevalence Ratios estimating factors associated with injury severity (ISS>9) in a multivariate logistic regression model (N = 1,656), Japan, 2003

Factors	N	PR adj.	95% CI	
Gender				
Females	744	1.00		
Males	912	1.54	1.04	2.28

Age				
18 - 64	1459	1.00		
65 - 74	119	3.96	2.35	6.65
75 -	78	2.44	1.32	4.51

Mechanism of Injury				
Automobile	1067	1.00		
Bicycle	182	4.36	2.52	7.55
Motorcycle	263	2.69	1.65	4.40
Pedestrian	111	3.57	3.11	4.02
Others	33	1.70	0.52	5.40

Address				
in Kurume	976	1.00		
outside Kurume	680	2.83	1.90	4.19

Transfer				
Walk-in	813	1.00		
Ambulance	843	21.79	9.47	50.14

Weekend				
Yes	585	1.00		
No	1071	1.79	1.18	2.72

PR adj: Adjusted Prevalence Ratios

The area under ROC curve = 0.86

P-value of the goodness of the fit = 0.925

CI: Confidence Intervals

## Discussion

### Main findings

As we hypothesized, injuries to people aged 65 and older were different from those experienced by younger adults in terms of both injury diagnosis and severity, as indicated by national and hospital-based data. Regarding mechanism of injury, although automobile incidents (drivers or occupants) were the main cause in this study population, the percentage of pedestrian and bicycle incidents increased with increasing age, in both national and hospital data. Richter and et al. reported similar findings [21]. Also, intrathoracic injury, intracranial injury, and lower extremity fractures were more common among people aged 65 and over than among younger people, likely associated with injuries from bicycles or pedestrians. Among all pedestrian and bicycle injuries, chest, head, and lower-extremity injuries were frequent, similar to findings reported by Demetriades et al., in a study of injury severity by age for pedestrians injured by automobile [22]. Hospital payment should be covered by mandatory care insurance in Japan; however, this study showed that more than 30% was paid by other types of insurance or self pay. Hospital stay days were not different from people aged 65-74 and those aged 75+.

The high percent of neck sprain and strain found in this study may be related to an overuse of this diagnosis by physicians in the ED as a "catch all" diagnosis when no other diagnosis fits [23-26]. Because Japan's universal health insurance system covers the medical cost of traffic injuries, there are not significant barriers to accessing hospital care, and people visit hospitals and clinics easily. We analyzed the data removing all the neck sprain/strain patients, and our results were the same for the three other leading traffic injuries for people aged 65 years and older.

Seeing the point estimates of PR in hospital admission and injury severity compared with reference, PRs for the patients aged 65-74 were slightly higher than those aged 75+, and there might be a possibility of different patterns of injury for people aged 65 - 74 than for people aged 75+. One possible explanation for the difference regarding high injury severity is that most people aged 65 - 74 in Japan are retired but still socially active, while at the same time their physical ability is declining gradually. As a result, there is a gap between what they would like to do and what they are able to do in daily life, which may be associated with traffic injury. Such partial impairment may place 65 - 74 year olds at risk for vehicle-related injuries and it will be important to study this observation further through in-depth interviews with this age group [21,22,27,28].

At the same time, in the multiple regression analysis, we used patients aged 75+ as a reference, and confirmed the associations with other age groups. The prevalence ratio (ISS ≥ 9) of age 65-74 and age 75+ was 1.620 (95%CI 0.790 - 3.321). Also, the prevalence ratio (hospital admission) of age 65-74 and age 75+ was 1.138 (95%CI 0.582 - 2.225). We confirmed there were not statistical differences between age 65-74 and age 75+ with respect to hospital admission and injury severity. This was partly because this observational study did not have adequate power to detect differences, and the interpretation should be done with care.

Higher injury severity scores rates were associated with older age, and these two factors combine to result in increased medical costs [21,25]. Considering that Japan's elderly population is increasing, that they are at risk for traffic injuries especially as pedestrians and that they appear to be more likely to sustain costly injuries, effective interventions to reduce this risk are needed.

For the purpose of distinguishing severity level within the study population, the definition of slight/moderate injuries was based on a low cut-off point (ISS greater than four), and there were a large proportion of cases with slight/moderate injuries. However, we believe that this categorization scheme has proven useful in focusing on the consequences of the most severe injuries [16,29,30].

### Limitations and Strengths

Some limitations exist in this study. First, the emergency department data do not represent population-based incidence, and selection bias should be taken into account. Because there are several hospitals with emergency departments in the area, in addition to Saint Mary's Hospital, all the traffic injuries that occurred in Kurume and the surrounding areas were not captured in our emergency department data. Some, but not all, severe injuries were likely seen at the Level 1 trauma centre in the university hospital located in Kurume. Second, the association between pre-existing diseases and their clinical injury consequences could not be investigated in the study because of unavailability of data. Third, the study data did not have adequate power to detect statistical differences, and a larger sample size is mandatory for further studies.

Alternatively, this study has several strengths. It is the first to document clinical consequences of road traffic injuries among the elderly using hospital emergency department registry data in Japan. Although the results are based on a single hospital and the sample size was small, standardized data collection, including ICD-10 injury coding, was used for making the data more reliable. If the same efforts are employed in multiple other hospital settings, more reliable results will be obtained.

### Implications

This study serves as an important first step in developing a hospital-based injury registry in Japan. It demonstrates that tracking emergency department injury-related visits in an active hospital in suburban Japan was feasible, given adequate hospital cooperation and proper training of personnel to review and code emergency department visits [11,16,31]. Further, the study contributes new knowledge about leading sources of traffic injuries in a suburban area of Japan, and particularly the identification of factors associated with risk of severity, admission, and traffic-related injury circumstances among the elderly [21].

Japan is thought to be successful in its elderly health practices and policies because of the long life expectancy of its population; however, there are many unsolved public health issues among the elderly, including road traffic injuries. The National Police Agency, Japan, has proposed that elderly people aged 75+ are more involved in traffic incidents [7,8]. However, the results of our study suggest further studies focused on people 65-74 years old should be done.

Finally, these data are important because they suggest factors associated with elderly traffic injuries in Japan that should be explored further. The next step in achieving a more complete picture of road traffic injuries among the elderly in Japan is a systematic registry that includes data from multiple hospital emergency departments.

## Conclusions

Based on hospital emergency department injury registry data in Japan, injury circumstances and nature of injury associated with traffic incidents among the elderly indicate different patterns by age groups.

## Competing interests

No known conflicts of interest pertaining to authors are pertinent to the publication of this report.

## Authors' contributions

All authors have contributed to each of three manuscript preparation activities: conception/design and analysis/interpretation; writing; and approval of the final version, and will take public responsibility for the content of the paper.

## Pre-publication history

The pre-publication history for this paper can be accessed here:

http://www.biomedcentral.com/1471-2458/10/375/prepub

## References

[B1] World Health Organization World report on road traffic injury prevention 2004 WHO, Geneva

[B2] Aschkenasy MT Rothenhaus TC Trauma and falls in the elderly Emerg Med Clin North Am 2006 24 413 32 10.1016/j.emc.2006.01.005 16584964

[B3] Centers for Disease Control and Prevention Older Adult Drivers: Fact Sheet Atlanta 2007

[B4] European Network for Safety among Elderly (EUNESE) Partners Five-Year Strategic Plan for the Prevention of Unintentional Injuries among EU Senior Citizens Athens 2006

[B5] Organization for Economic Co-operation and Development Aging and Transport. Mobility needs and safety issues Paris 2001 40 41

[B6] Ministry of Health, Labor and Welfare The fact sheet for longevity in Japan Tokyo 2009 (in Japanese)

[B7] Information for Traffic Accident Research and Data Analysis Web-based database for traffic crashes in Japan 2009 http://www.itarda.or.jp/english/Accessed July 1, 2010.

[B8] Japan National Police Agency The Police Report about fatal traffic crashes in 2007 2008 Japan Tokyo (in Japanese)

[B9] Laflamme L Svanström L Schelp L Safety Promotion Research. A Public Health Approach to Accident and Injury Prevention 1999 Stockholm: Karolinska Institutet 63 82

[B10] McCaig LF Nawar EW National Hospital Ambulatory Medical Care Survey: 2004 emergency department summary Adv Data 2006 1 29 16841785

[B11] Perry MJ Sun BX Zhang HX Emergency department surveillance of occupational injuries in Shanghai's Putuo District, People's Republic of China Ann Epidemiol 2005 15 351 7 10.1016/j.annepidem.2004.12.007 15840548

[B12] World Health Organization Guidelines for conducting community surveys on injuries and violence 2004 WHO, Geneva 10.1080/156609704/233/327505 15903167

[B13] World Health Organization Holder YMP Krug E Lund J Gururaj G Kobusingye O Injury surveillance guidelines 2004 WHO, Geneva 11 17

[B14] Nakahara S Wakai S Underreporting of traffic injuries involving children in Japan Inj Prev 2001 7 242 4 10.1136/ip.7.3.242 11565993 PMC1730737

[B15] Association for the Advancement for Automotive Medicine, Committee on Injury Scaling The Abbreviated Injury Scale, 1990 Revision (AIS-90) 1990

[B16] Cirera E Plasencia A Ferrando J Segui-Gomez M Factors associated with severity and hospital admission of motor-vehicle injury cases in a southern European urban area Eur J Epidemiol 2001 17 201 8 10.1023/A:1017961921607 11680536

[B17] Plasencia A Borrell C Anto JM Emergency department and hospital admissions and deaths from traffic injuries in Barcelona, Spain. A one-year population-based study Accid Anal Prev 1995 27 591 600 10.1016/0001-4575(95)00007-M 7546070

[B18] Thompson ML Myers JE Kriebel D Prevalence odds ratio or prevalence ratio in the analysis of cross sectional data: what is to be done? Occup Environ Med 1998 55 272 7 10.1136/oem.55.4.272 9624282 PMC1757577

[B19] Zocchetti C Consonni D Bertazzi PA Relationship between prevalence rate ratios and odds ratios in cross-sectional studies Int J Epidemiol 1997 26 220 3 10.1093/ije/26.1.220 9126523

[B20] Hosmer DW Lemeshow S Applied Logistic Regression 2000 Chapter 5 Second New York: John Wiley & Sons 147 156

[B21] Richter M Pape HC Otte D Krettek C The current status of road user injuries among the elderly in Germany: a medical and technical accident analysis J Trauma 2005 58 591 5 10.1097/00005373-200503000-00024 15761356

[B22] Demetriades D Murray J Martin M Velmahos G Salim A Alo K Rhee P Pedestrians injured by automobiles: relationship of age to injury type and severity J Am Coll Surg 2004 199 382 7 10.1016/j.jamcollsurg.2004.03.027 15325607

[B23] Hijioka A Narusawa K Nakamura T Risk factors for long-term treatment of whiplash injury in Japan: analysis of 400 cases Arch Orthop Trauma Surg 2001 121 490 3 10.1007/s004020100284 11599748

[B24] Ono K Kanno M Influences of the physical parameters on the risk to neck injuries in low impact speed rear-end collisions Accid Anal Prev 1996 28 493 9 10.1016/0001-4575(96)00019-X 8870776

[B25] Ottosson C Nyren O Johansson SE Ponzer S Outcome after minor traffic accidents: a follow-up study of orthopedic patients in an inner-city area emergency room J Trauma 2005 58 553 60 10.1097/01.TA.0000152634.66513.AF 15761351

[B26] Schrader H Obelieniene D Bovim G Surkiene D Mickeviciene D Miseviciene I Sand T Natural evolution of late whiplash syndrome outside the medicolegal context Lancet 1996 347 1207 11 10.1016/S0140-6736(96)90733-3 8622449

[B27] Oxley J Fildes B Ihsen E Charlton J Day R Differences in traffic judgments between young and old adult pedestrians Accid Anal Prev 1997 29 839 47 10.1016/S0001-4575(97)00053-5 9370020

[B28] Oxley JA Ihsen E Fildes BN Charlton JL Day RH Crossing roads safely: an experimental study of age differences in gap selection by pedestrians Accid Anal Prev 2005 37 962 71 10.1016/j.aap.2005.04.017 15993827

[B29] MacKenzie EJ Siegel JH Shapiro S Moody M Smith RT Functional recovery and medical costs of trauma: an analysis by type and severity of injury J Trauma 1988 28 281 97 10.1097/00005373-198803000-00003 3351987

[B30] MacKenzie EJ Steinwachs DM Shankar B Classifying trauma severity based on hospital discharge diagnoses. Validation of an ICD-9CM to AIS-85 conversion table Med Care 1989 27 412 22 10.1097/00005650-198904000-00008 2649755

[B31] Ferrando J Plasencia A Ricart I Canaleta X Segui-Gomez M Motor-vehicle injury patterns in emergency-department patients in a south-European urban setting Annu Proc Assoc Adv Automot Med 2000 44 445 58 11558100 PMC3217388

